# Th17 Cells as Potential Probiotic Therapeutic Targets in Inflammatory Bowel Diseases

**DOI:** 10.3390/ijms160920841

**Published:** 2015-09-01

**Authors:** Eddy Owaga, Rong-Hong Hsieh, Beatrice Mugendi, Sakhile Masuku, Chun-Kuang Shih, Jung-Su Chang

**Affiliations:** 1 Institute of Food and Bioresources Technology, Dedan Kimathi University of Technology, 10100 Nyeri, Kenya; E-Mails: eowaga@yahoo.co.uk (E.O.); beatrice.mugendi@dkut.ac.ke (B.M.); 2 School of Nutrition and Health Sciences, College of Public Health and Nutrition, Taipei Medical University, 250 Wu-Xing Street, 110 Taipei, Taiwan; E-Mails: hsiehrh@tmu.edu.tw (R.-H.H.); ckshih@tmu.edu.tw (C.-K.S.); 3 Community Health Nursing Department, Faculty of Health Sciences, University of Swaziland, H100 Mbabane, Swaziland; E-Mail: SMasuku@uniswa.sz

**Keywords:** Th17, IL-17, probiotics, inflammatory bowel diseases, inflammation

## Abstract

Inflammatory bowel diseases (IBD) are characterized by wasting and chronic intestinal inflammation triggered by various cytokine-mediated pathways. In recent years, it was shown that T helper 17 (Th17) cells are involved in the pathogenesis of IBD, which makes them an attractive therapeutic target. Th17 cells preferentially produce interleukin (IL)-17A–F as signature cytokines. The role of the interplay between host genetics and intestinal microbiota in the pathogenesis of IBD was demonstrated. Probiotics are live microorganisms that when orally ingested in adequate amounts, confer a health benefit to the host by modulating the enteric flora or by stimulating the local immune system. Several studies indicated the effectiveness of probiotics in preventing and treating IBD (ulcerative colitis, and Crohn’s disease). Furthermore, there is mounting evidence of probiotics selectively targeting the Th17 lineage in the prevention and management of inflammatory and autoimmune diseases such as IBD. This review highlights critical roles of Th17 cells in the pathogenesis of IBD and the rationale for using probiotics as a novel therapeutic approach for IBD through manipulation of Th17 cells. The potential molecular mechanisms by which probiotics modulate Th17 cells differentiation and production are also discussed.

## 1. Introduction

Inflammatory bowel diseases (IBD) are characterized by chronic inflammation of the gastrointestinal (GI) tract. The global prevalence of IBD ranges 12%–20% [1]. The two major types of IBD include Crohn’s disease (CD, inflammation of any section of gastrointestinal tract), and ulcerative colitis (UC, inflammation of the lining of the large intestine) [2]. In addition, pouchitis describes the inflammation of the intestinal wall of the ileal reservoir, which is associated with colectomy and restorative surgery [2]. The pathogenesis of IBD is mainly linked to uncontrolled mucosal inflammation due to an abnormal immune response against luminal antigens and microbiota [3]. This response is mainly triggered by a breakdown in the intestinal homeostasis among the microbiota, intestinal epithelial cells, and resident immune cells [4]. Innate and adaptive immune responses are important factors in the development of IBD [5]. In the early phase of inflammation, innate immune cells, including mast cells, neutrophils, macrophages and dendritic cells, located in the intestine are recruited in response to antigens and commensal bacteria. Subsequently, the persistent inflammation arising from the released proinflammatory innate immune cells results in activation of an adaptive immune response [4]. Both innate and adaptive immune responses result in chronic tissue injury and epithelial damage related symptoms in IBD patients [1,4].

Numerous previous studies showed that CD4^+^ T helper (Th)1 and Th2 cells are essential in the pathogenesis of IBD [6,7]. CD and UC are widely associated with Th1 cytokines (tumor necrosis factor (TNF)-α; interferon (IFN)-γ; interleukin (IL)-12) and Th2 cytokines (IL-5 and IL-13) in the mucosa [4,5]. Nevertheless, despite extensive research on the roles of Th1 and Th2 cytokines, knowledge of factors causing IBD remains incomplete. Recently, a new distinct subset of Th17 cells capable of producing IL-17 was associated with the IBD pathogenesis due to their proinflammatory roles in the mucosal immune response [4,6]. Secreted IL-17 exists in six isoforms: IL-17A–F [4,6]. In addition to the IL-17 isoforms, Th17 cells also differentiate and secrete other proinflammatory cytokines such as IL-6, IL-1β, TNF-α, IL-12, tumor growth factor (TGF)-β, IL-23, IL-21, IL-22, and IL-26 during the IBD pathogenesis [6].

Several human studies demonstrated the pathogenic role of Th17 in IBD. According to Sartor [8], IL-17 messenger (m)RNA is highly expressed in inflamed mucosa obtained from both UC and CD patients. In other comparative studies, the number of Th17 cells and IL-17 expression were markedly enhanced in the inflamed gut of CD and UC patients [9,10]. In addition, elevated levels of IL-17 were reported in intestinal tissues and serum of IBD patients [11]. Further evidence of the role of Th17 in IBD development is the strong correlation between the disease severity and IL-17 levels in speripheral blood mononuclear cells from UC patients [12]. Moreover, in a cohort study of subjects with CD and UC, patients with IBD showed a remarkably higher prevalence of circulating IL-17 than did the control group [10].

Several animal studies also showed etiological implications of IL-17A in IBD pathogenesis. Ito *et al.* [13] observed diminished manifestations of colitis in mice with a deficiency of the IL-17A gene after administration of dextran sodium sulfate (DSS). In addition, Zhang *et al.* [14] also reported that IL-17R-knockout mouse were significantly protected against colonic inflammation in a model of trinitrobenzenesulfonic acid (TNBS)-induced colitis. Taken together, these observations imply that Th17 cytokines are strongly linked to the pathogenesis of IBD and hence are an attractive therapeutic target for IBD prevention and management.

The primary aim of this review is to highlight critical roles of Th17 cells in the pathogenesis of IBD and the rationale for using probiotics as a novel therapeutic approach through manipulation of Th17 cells in IBD. Specifically, this review examines the modulatory role of probiotics on Th17 in IBD *in vitro*, and in animal and human studies, and provides insights into the potential underlying molecular mechanisms of probiotic modulation of Th17 towards alleviating IBD.

## 2. Role of Th17 Cells in the Gut Immune Homeostasis

Roles of Th17 cells in intestinal pathology and homeostasis still remain poorly understood. Nevertheless, as outlined below, some of the suggested roles in gut-related tissues include; (i) promotion of microbial defense; (ii) modulation of T cell differentiation and cellular production of inflammatory mediators [6]; and (iii) modulation of neutrophil migration and function.

Although controversial, it has been suggested that Th17 cells may confer a protective effect on the GI tract under steady-state homeostatic regulation, but initiate an uncontrolled pathogenic immune response during dysbiosis [5,15,16,17,18]. Th17 cells are particularly believed to clear fungal and extracellular bacterial infections that are not efficiently cleared by Th1- and Th2-type immunity [5,19]. IL-17A production has been reported upon infection with *Staphylococcus aureus*, *Clostridium rodentium*, *Klebsiella pneumonia*, *Mycobacterium tuberculosis*, *Leuconostoc monocytogenes*, *Salmonellae typhimurium*, and *Pneumocystic carinii* [20]. IL-17A was also shown to fortify tight junction formation between epithelial cells by inducing expressions of claudins and by stimulating mucin production by intestinal epithelial cells, thereby increasing the integrity of the intestinal barrier [21,22]. In humans, the suppression of Th17 production due to defective *STAT3* gene mutation has been linked to hyper-immunoglobulin E syndrome (HIES). HIES is characterized by abnormal susceptibility to *S. aureus*, *S. pneumonia*, and *C. albicans* infections [20]. In animal studies, a protective role was suggested for IL-17A in a T-cell transfer model of colitis [23,24]. In agreement with this observation, treatment of mice with an anti-IL-17 neutralizing antibody enhanced the severity of DSS-induced colitis [22]. It was also revealed that murine DSS-induced colitis was worsened in IL-17A-knockout mice but was substantially improved in IL-17F-knockout mice [25]. These contrasting results on the pathogenic and protectivde roles of Th17 are, at least partly, attributed to differences in ligand affinity, downstream signalling cascades, and receptor tissue distribution [4] and other factors that influence the action of Th17 and may be present in the local environment [5].

Notably, of critical relevance to this review is the pathogenic role of Th17 in IBD. Th17 cells are involved in the priming and differentiation of proinflammatory cytokines in the IBD pathogenesis. After activation, Th17 cells secrete various isoforms of cytokine IL-17 (IL-17A–F), which eventually stimulate intestinal endothelial cells, myofibroblasts, and epithelial cells to produce other proinflammatory mediators [14,23]. Furthermore, several studies demonstrated that Th17 cells can directly induce the secretion of several proinflammatory effector molecules which mediate tissue infiltration and tissue destruction, including proinflammatory cytokines (IL-6, IL-1β, TNF-α, IL-12, TGF-β, IL-23, IL-21, IL-22, and IL-26), chemokines, and matrix metalloproteases [4,5,6,26]. The imbalance in the microbiota profile in the gut, can transform the steady “peace” state immune status in healthy individuals to activated “war” state characterized by rapid expansion of Th17 cells [4]. For instance, proliferation of pathogens provides Toll-like receptor (TLR) ligands that activate dendritic cells to produce IL-6 and TGF-β, which subsequently promote differentiation of Th17 cells. Uncontrolled production of Th17 cells can lead to dysregulated production of pro-inflammatory cytokines and chronic inflammation, eventually contributing to tissue damage in IBD [20]. For this reason, modulation of the differentiation and function of Th17 cells is currently viewed as a potentially feasible therapeutic target for intestinal inflammatory conditions experienced in IBD.

Emerging data suggests IL-17 can influence neutrophil migration and function during inflammation. Grifin *et al.* [27] found that IL-17 enhanced expression of neutrophilic chemokines CXCL1, CXCL2, and CXCL5 through endothelial activation. Taylor *et al.* [28] also reported human and mouse neutrophils that were produced and responded to IL-17A. In animal models, endogenous IL-17 mediated the recruitment of neutrophils in mouse airways upon endotoxin exposure [29].

## 3. Risk Factors for the Development of IBD

The pathogenesis of IBD has been associated with interaction of multiple factors, namely: (i) genetic susceptibility factors; (ii) host immunity homeostasis; (iii) integrity of the intestinal epithelial cells; and (iv) environmental factors, such as microbiota [30,31,32].

### 3.1. Genetic Susceptibility Factors

Generally, IBD arise from a disruption of mucosal immune homeostasis in genetically susceptible individuals [5,33]. A recent genome-wide study on the role of Th17 cells in the pathogenesis of IBD showed that genes involved in Th17 differentiation are associated with a susceptibility to IBD [2,34]. On the other hand, it was found that a mutation of the gene encoding the IL-23 receptor (IL-23R) is strongly associated with IBD [35]. Siakavellas and Bamias [36] also identified several CD-associated polymorphisms in genes that encode for proteins of the IL-23/Th17 pathway. Other genome-wide association studies also revealed polymorphisms in additional genes that are linked to a risk of IBD pathogenesis. Some of the genes implicated in the onset of both CD and UC are essential for intestinal homeostasis, including T cell regulation (*TNFSF8*, *IL12B*, *IL23R*, *PRDM1*, and *ICOSLG*), immune tolerance (*IL10* and *CREM*), innate mucosal defense (*CARD9* and *REL*), restitution (*REL*, *PTGER4*, and *NKX2-3*), paneth cells (*XBP1*), and immune cell recruitment (*MST1*) [2]. Bogoaert *et al.* [37] studied differential mucosal expression of genes involved in differentiation (*IL-6*, *IL-1β*, *TGF-β*, *IL-23A* and *STAT3*) and recruitment of Th17 cells (*CCR6* and *CCL20*) and observed enhanced expression of these genes in ileal and colonic samples from UC and CD patients. Seiderer *et al.* [38] reported increased colonic *IL17F* gene expression in active CD.

### 3.2. Host Immunity Homeostasis

The gastrointestinal tract regulates immune responses of the immune system against pathogens and commensals, thereby maintaining homeostasis of the human gut [4]. It is generally accepted that chronic intestinal inflammation and damage arise from excessive and uncontrolled mucosal immune responses (particularly by CD4^+^ T cells) against commensal microbes in the gut in individuals with a genetic susceptibility [6,18]. Traditionally, CD and UC have been associated with Th1 cytokines (TNFα, IFNγ, IL-12), and Th2 cytokines (IL-5, IL-13), respectively [5]. In addition, inflammation in UC is continuous in the colon whereas inflammation in CD may lead to inflamed sections mixed with healthy parts of intestine [4].

### 3.3. Integrity of Intestinal Epithelial Cells (IECs)

IECs are important components of the intestinal barrier between mucosal immune cells and luminal tissues, which are crucial in maintaining the immune homeostasis of the gut, by physically separating commensal bacteria and mucosal immune cells [3]. In addition, IECs integrate microbial signals into the mucosal immune system through pattern-recognition receptors [30]. Using a specific Ripk1 mice model, it was established that mice developed severe intestinal inflammation due to IEC death and subsequent dysfunction of the intestinal barrier function [39]. To further support the role of IECs in the pathogenesis of IBD. Dotan *et al.* [34] found that IECs from IBD patients excessively induced CD4^+^ T cell proliferation and produced interferon (IFN)-γ due to disturbed intestinal immune homeostasis. Overall, those studies revealed that alterations of the integrity of IECs drive chronic intestinal inflammation, and hence controlling IECs is essential to prevent uncontrolled mucosal inflammation.

### 3.4. Environmental Factors such as Microbiota

The role of the gut microbiota in the pathogenesis of human IBD was highlighted by several authors [4,40]. Interactions of microbes with mucosal immune cells in the GI tract are important in priming and regulating immunity [41]. Thus, increased evidence showed that IBD primarily represent an imbalance of the intestinal microbiota and a malfunction of tolerance to commensal microbiota [42]. Changes in the commensal bacterial floral composition within the GI tract are responsible for altering immune responses from a steady state to activated, aggressive, and damaging immune responses characterized by expansion of Th17 cells [31]. There is mounting evidence that normal microbiota such as *Lactobacillus* spp. and *Bifidobacteria* spp. in the GI tract are significantly reduced in patients with IBD [43,44] but instead are associated with susceptibility to pathogens such as *Clostridium difficile* [45], Actinobacteria and Proteobacteria [46], and *Streptococcus* spp. [47]. According to Gálvez [4] pathogen-infected epithelial cells provide Toll-like receptor (TLR) ligands, leading to secretion of IL-6 and TGF-β from dendritic cells, hence activating Th17 differentiation in IBD.

## 4. Potential Therapeutic Roles of Probiotics in IBD

In the recent past, there was intense research on developing new therapeutics, such as probiotics, which can reduce intestinal inflammation and restore a balance to the GI tract microbiota in IBD. Probiotics are defined as living microorganisms which when consumed in adequate amounts confer health benefits to the host beyond inherent general nutrition [17,48]. However, a considerable number of recent studies indicated that beneficial effects can also be achieved by heat-inactivated probiotics, isolated bacterial DNA, or probiotic-cultured media [49,50]. Lactobacilli and bifidobacteria are predominantly present in the ileum and colon, and hence both have been widely studied for preventing and managing IBD [17,31,51,52].

The efficacy of probiotics in IBD has been examined in various animal models and clinical studies, and respective outcomes were reviewed by several authors [1,3,31,41,51,53,54]. Despite a few cases of conflicting results, probiotics have generally successfully been demonstrated to have some efficacy in alleviating inflammation in human IBD and murine colitis models. Several clinical studies indicated the effectiveness of specific probiotic strains towards reduction of UC severity. These include *L. casei* subsp. *rhamnosus* [55]; *Bifidobacteria breve*, *B. bifidum*, *L. acidophilus* [56]; VSL#3 (mixture of *L. plantarum*, *acidophilus*, *L. casei*, *L. delbruecki* subsp. *bulgaricus*, *B. longum*, *B. infantis*, *B. breve* and *Streptococcus thermophilus*) [57]. In contrast, other studies have shown no significant benefit of *E. coli* Nissle 1917 [58] and a mixture of *L. salivarius* and *B. infantis* [45]. Several clinical trials have also demonstrated efficacy of probiotics in alleviating inflammation in CD. These include VSL#3 [59], and *S. boulardii* [60]. However, other clinical trials have reported no effect of some probiotics in preventing CD such as *L. rhamnosus* LGG [61,62,63,64], and *L. johnsonii* LA1 [65,66]. Some clinical studies have shown beneficial effect of probiotics on pouchitis including VSL#3 [67,68] and *L. rhamnosus* LGG [69]. In contrast, other comparative studies have shown no significant effect of VSL#3 [70] and *L. rhamnosus* LGG [71] on pouchitis compared to respective controls. Several studies conducted using murine models have demonstrated the effectiveness of probiotics in alleviating TNBS- or DSS-induced colitis. These include *L. fermentum* [50,72,73,74], *L. acidophilus* [75], *B. longum* subsp. *infantis* JCM 1222 [32] and *S. thermophilus* ST28 [17].

As suggested by various authors [31,76], inconsistencies in outcomes of some clinical trials of IBD can be explained in several ways: (i) marked heterogeneity between IBD trials with regard to choices of probiotic and their dose, the trial design, and outcome measures evaluated; (ii) variations in the populations studied, with some trials enrolling patients with active disease; (iii) most studies having enrolled small numbers of patients, which limits the statistical power and may account for the high placebo response rates reported in IBD clinical trials; and (iv) most studies not indicating details of the patients’ diets, which may potentially influence the efficacy of the probiotics.

## 5. Mechanisms Underlying Probiotic Anti-Inflammatory Effects on IBD Pathogenesis

Although it is apparent from animal and human studies that probiotics may confer relief of IBD symptoms to the host upon consumption, the exact mechanisms by which probiotics impart protective effects are still not fully understood. As briefly outlined below, multiple broad mechanisms have been suggested including: (i) displacement and suppression of the growth of pathogens [17]; (ii) improved epithelial barrier function [32,50]; and (iii) immunomodulation of Th1, Th2, regulatory T cell, and Th17 cell production [17].

### 5.1. Displacement and Suppression of Growth of Pathogens

Probiotics are believed to rapidly colonize the gastrointestinal tract hence competitively inhibit pro-adherence of pro-inflammatory pathogens to the IEC [31,32]. Several invasive pathogenic bacteria such as *E. coli* and *C. difficile* are associated with IBD development [77,78]. A number of human studies have demonstrated that after administration of VSL#3 probiotic mixture, the relief of the inflammation conditions in IBD patients are accompanied by increased colonization of the gastrointestinal tract [64,79]. Administration of *L. acidophilus* also led to increased lactobacilli and bifidobacteria profile but reduced *Staphylococcus aureus* population in the distal colon section [75]. In murine models, some authors reported that *L. fermentum* ACA-DC 179 [73] and Duolac Gold (mixture of lactobacilli bacteria) [48] inhibited the growth of Salmonella and *C. difficile*, respectively.

### 5.2. Improved Epithelial Barrier Function

Several studies have demonstrated that the metabolites produced by bacteria may interact directly with gut epithelial cells to enhance mucosal barrier function [80]. Peran *et al*. [50] reported that heat-killed *L. brevis* SBC8803 ameliorates intestinal injury in a murine model of colitis by enhancing the intestinal barrier function. The exact mechanisms underlying improved epithelial barrier function are yet to be elucidated.

### 5.3. Immunomodulation of Th1, Th2, Regulatory T Cells, and Th17 Cells Production

In the intestinal tract, immunocytes such as M cells and dendritic cells are constantly responding to intestinal bacteria, an event referred to as microbe-host “cross-talk” [40,51,81]. Consequently, upon consumption, probiotic bacteria or cell wall components are internalized by M cells to interact with dendritic cells, which further influence the polarization of T-cell responses (Th1, Th2, or regulatory T cells) [40]. Therefore, it is generally recognized that the anti-inflammatory effect of probiotics in IBD is linked to the down-regulation of pro-inflammatory IL-17 cytokine production and upstream Th-17 secreted cytokines (IL-23, TGF-β) and downstream Th-17 secreted cytokines (IL-1β, IL-6, TNF-α, IFNγ, IL-12).

#### 5.3.1. Immunomodulation of IL-17 and IL-23 Cytokines

Several animal experiments and clinical trials have demonstrated that anti-inflammatory effects of probiotics in IBD might be a consequence of the down regulation of pro-inflammatory IL-17 production [17,26]. Various studies on colitis-induced animal models have revealed several probiotics that can down regulate IL-17 production as well as simultaneously alleviate colitis. These probiotics include *B. breve* [33], *B. longum* [32], *L. acidophilus* [6], *B. longum* subsp. *infantis* [26], *S. thermophilus* ST28 [82], and *L. gasseri* A5 [33]. The feasibility of the proposed mechanism of probiotic inhibition of IL-17 target is supported by a comparative study showing 4SC-101 [83] and Vidofludimus [84], both novel immunosuppressive drugs, inhibit IL-17 and attenuates chronic colitis in mice. IL-23 is responsible for expansion, stabilization and conditioning of Th17 hence its key role in the activation of inflammation in IBD [6]. The IL-23/17 axis is widely regarded as a hallmark of IBD and is therefore an attractive probiotic therapeutic target in IBD prevention and management [18,33,85]. Indeed, Ghadimi *et al.* [33] investigated the effect of *B. breve* and *L. rhamnosus* GG on the expression of IL-23 on intestinal cells and found diminished liposaccharide-induced expression of IL-23.

#### 5.3.2. Immunomodulation of TGF-*β*, IL-1*β* and IL-6 Cytokines

TGF-*β* regulates Th17 differentiation by acti*β*ating transcription factor signal transducer for transcriptor 3 (STAT3) [86]. Kumar *et al.* found reduced expression and production of TGF-*β* in colitis mouse models after administration of probiotic mixture comprising four *lactobacilli* and eight *Bifidobacterium* spp. [87]. IL-1*β* is critical for induction differentiation and development of Th17 [88]. Reduced expression of IL-1*β* in the colon have been found in colitis mouse models after oral administration of *L. brevis* SBC 8803 [89], and lactobacilli mixture [90]. IL-6 has been shown to initiate Th17 differentiation in IBD pathogenesis. Several studies have shown reduced levels of IL-6 expression and production as well as parallel relief of intestinal damage in DSS- and TNBS-induced colitis mice models after administration of probiotics. These include *L. fermentum* CECT 5716 [74], Duolac Gold (mixture of seven species of bifidobacteria, lactobacilli bacteria and *S. thermophilus*) [48], and lactobacilli mixture [90].

#### 5.3.3. Immunomodulation of TNF-α and IFNγ Cytokines

TNF-α is among the pro-inflammatory cytokines secreted downstream of Th17 cells differentiation during IBD pathogenesis. Several authors have reported the ability of several probiotics to down regulate the expression and production of TNF-α in DSS- and TNBS-induced colitis mouse, namely *L. brevis* SBC 8803 [89], mixture of lactobacilli and bifidobacteria [91], *L. fermentum* [50], *B. lactis* [92], *L. salivarius* subsp. *salivaris* [93] and probiotic mixture of four *lactobacilli* spp. and eight *Bifidobacterium* spp. [87]. IFNγ is secreted downstream of Th17 activation hence contribute to inflammation in IBD. Several studies in mouse models of induced colitis have shown probiotics can inhibit expression and production of IFNγ. These include lactobacilli and bifidobacteria mixture [91], and lactobacilli mixture [90].

#### 5.3.4. Immunomodulation of IL-12 and IL-10 Cytokines

IL-12 production also occurs downstream of Th17 activation in IBD. Some studies have shown probiotic s such as *L. brevis* SBC 8803 [89], and *lactobacilli* and *bifidobacterium* spp. mixture [91] can inhibit expression and production of IL-12 in colitis mouse models. The down-regulation of Th17 cells production by probiotics also has been associated with the up-regulation of anti-inflammatory cytokines such as IL-10, which are vital in the maintenance of the immune balance. Several studies on anti-inflammatory potential of probiotics have shown increased expression and production levels of IL-10 after administration to colitis mouse models. These include *B. longum* subsp. *infantis* JCM 1222T [26], *L. salivarius* Ls 33, *E. coli* Nissle 1917 [94], *L. plantarum* [95], *L. rhamnosus* GG, *S. thermophilus*, *B. animalis* subsp. *lactis*, *B. breve* [96], VSL#3 mixture [97], lactobacilli and bifidobacteria mixture [91] and *L. fermentum* [73]. The IL-10/IL-12 ratio is relatively low in IBD patients and studies have shown that some probiotics such as VSL #3 mixture have a potential of inducing IL-10 production in IBD subjects [17]. In addition, high IL-10/IL-12 ratio, a common indicator of the probiotic anti-inflammatory effect potential, was induced after administration of *B. longum* subsp. *infantis* [98].

Overall, the discrepancies on the beneficial effects between the various probiotic species and strains, have largely been attributed to the differences in the cell wall structure of the probiotic strains especially the microbe-associated molecular patterns such as lipoteichoic acid, peptidoglycan, and non-methylated CpG motifs [75]. Due to the limited number of human studies, most of the hypotheses on IBD pathogenesis have been assessed using mouse models, which have led to improved understanding of the mechanisms underlying pathogenesis of IBD [99]. However, it is important to interpret the mouse model results with caution because of the emerging concerns on the variations in the origin and plasticity of human and murine Th17 cells [100].

## 6. Potential Molecular Mechanisms for Th17 Suppression by Probiotics

The mechanisms by which probiotics modulate Th17 cell differentiation and production in the gut still remain to be fully elucidated. As briefly described below, some of the emerging potential mechanisms include: (i) inhibition of co-stimulatory molecules (CD40 and CD80) in IECs; (ii) downregulation of Th17 cell transcription factors retinoic-acid-related orphan receptor (ROR)γt, STAT3, and nuclear factor (NF)-κB; and (iii) modulation of TLR signaling and production of extracellular ATP in lumen propria.

### 6.1. Inhibition of Co-Stimulatory Molecules in IEC (CD40, CD80)

As antigen-presenting cells, inflamed IECs could function by expressing higher levels of costimulatory molecules, which cause abnormal activation of T cells [101,102]. Indeed, using mouse intestinal epithelial cell line Colon-26 cells, it was demonstrated that inflamed IECs induce IL-17A response via co-stimulatory molecules CD80/CD86 and CD40 [103]. Thus, these molecules are considered as important IBD-causing factors and therefore potential probiotic therapeutic target for IBD. In this regard, *B. longum* subsp. *infantis* JCM 1222 has been shown to directly act on IECs to suppress CD80 and CD40 expression, and subsequently resulting in the suppression of mRNA and protein expression levels of IL-17A and alleviation of colitis [32]. In another comparative study, *B. breve* and *L. rhamnosus* GG suppressed the liposaccharide-induced mRNA expression of CD40 in an *in vitro* model of the human intestinal HT-29/B6 or T84 cells mucosal immune system [33]. As a whole, these modulatory effects give an insight on the novel therapeutic role of probiotics in alleviating IBD via suppression of the costimulatory molecules in IECs such as CD80/CD86 and CD40, which are vital in activating expression of IL-17A during IBD pathogenesis.

### 6.2. Down-Regulation of Th17 Cell Transcription Factors RORγt, STAT3, and NF-ĸB

Retinoic-acid-related orphan receptors (RORγt), has been identified as the transcription factor for Th17 cells [4,5]. Indeed, Ivanov [104] did not observe Th17 in RORγt-deficient mice, but the production of Th17 was induced upon transduction of naive T cells with a RORγt­encoding retrovirus, hence demonstrating the integral role of RORγt in Th17 cells differentiation. Thus, it has been proposed that RORγt could be potential probiotic therapeutic target towards inhibition of Th17 cells in IBD [10]. Subsequently, upon oral administration of *B. longum* subsp. *infantis*, [32] observed suppressed production of Th17-cytokines (IL-17A), which was parallel to down-regulated expression of RORγt.

The NF-ĸB and STAT3 signaling pathway is critical in promoting expression of pro-inflammatory Th-17 cell secreted cytokines, leading to intestinal inflammation in IBD pathogenesis [4,18,105]. In addition, transcription factor STAT3 has been reported to regulate the expression of the transcription factor RORγt, which is critical in Th17 differentiation and development [6]. To demonstrate the role of STAT3 in Th17 development, Galvez [4] reported that STAT3 overexpression promoted Th17, but the process was impaired in mice with STAT3-deficient cells. Furthermore, genome wide association studies have revealed polymorphism in Th-17 related genes such as STAT3 in IBD patients [2,33]. Therefore modulation of both the NF-ĸB and STAT3 mediated transcriptions of IBD—Causing Th-17 cell factors is viewed as a novel probiotic therapeutic target in prevention and management of IBD [18,33]. Indeed, Ghadimi *et al*. [106] reported that *B. longum* suppressed the activation of epithelial NF-ĸB via Toll-like receptor 9 in a colitis mouse model. Comparative studies *B. breve* and *L. rhamnosus* GG have also shown reduced nuclear translocation of NF-ĸB in human intestinal HT-29/B6 T84 cells [33]. Other probiotic bacteria that have exhibited suppressed activation of NF-ĸB in colitis mouse models include: *L. plantarum* HY115 and *L. brevis* HY7401 mixture [90], *B. breve*, *S. thermophilu*s, and *B. bifidum* mixture [107]. In addition, a clinical trial of IBD patients showed *Bifidobacterium* supplements reduced the expression of the pro-inflammatory transcription factor NF-ĸB. Regarding probiotic effect on STAT3 transcription, a study by Chen *et al*. [6] demonstrated *L. acidophilus* suppressed IL-17 production through down regulation of mRNA expression and phosphorylation of STAT3 [6]. Further, to support the novel role of NF-ĸB and STAT3 as potential therapeutic targets in prevention and treatment of IBD, Fitzpatrick [18] reported that Vidofludimus, an antibody drug, could inhibit IL-17 secretions in activated splenocytes by inhibiting STAT3 and NF-ĸB signaling pathways.

### 6.3. Modulation of TLR Signaling and Production of Extracellular ATP in Lumen Propria

Recent research advances by Atarashi *et al*. [108], show that production of Th17 cells in the intestinal lamina propria is, at least partly, regulated by the commensal bacteria. The two main hypothesized mechanisms include modulation of TLR and extracellular ATP [109]. Commensal bacteria release TLR ligands which stimulate lamina propria dendritic cells leading to differentiation of Th17 cells. In addition, commensal bacteria release extracellular ATP, which activate the dendritic cells to induce production of IL-6 and TGF-β cytokines leading to differentiation of Th17 cells.

## 7. Conclusions

More data from animal experiments and clinical trials support the potential therapeutic role of probiotics in IBD. Generally, the efficacy of probiotics in IBD is more evident in UC than in CD. Increasing evidence shows a new distinct subset of T helper cells (Th17), that are capable of producing IL-17 cytokines, plays key pathogenic roles in IBD due to their proinflammatory role in the mucosal immune response. The anti-inflammatory effects of probiotics in IBD might be a consequence of downregulation of IL-17 production and related proinflammatory Th17-secreted cytokines such as IL-23, TGF-β, IL-1β, IL-6, TNF-α, IFN-γ, and IL-12. Some of the emerging potential molecular mechanisms for the probiotics modulation of Th17 cell differentiation and production in the gut include inhibition of co-stimulatory molecules (CD40 and CD80) in IECs; downregulation of the Th17 cell transcription factors, RORγt, STAT3, and NF-ĸB; and modulation of TLR signaling and production of extracellular ATP in lumen propria. Further studies are required to elucidate which bioactive components from probiotic bacteria are responsible for the anti-inflammatory effects in IBD. Moreover, some critical differences appear to exist with regard to the pathogenic roles of various IL-17 isoforms in IBD; therefore, further studies should explore how probiotics can selectively alter expressions and production of specific IL-17 isoforms. It is anticipated that novel anti-inflammatory mechanisms hereby highlighted will improve our knowledge of how probiotics alleviate IBD pathogenesis and thus lead to the efficient identification and selection of beneficial probiotic strains with the goals of preventing and managing IBD symptoms.
